# Late-onset implant-related neuropathy: Three years after proximal humeral fracture

**DOI:** 10.1016/j.tcr.2022.100670

**Published:** 2022-06-28

**Authors:** Yasuaki Yamakawa, Yusuke Kamatsuki, Toshiyuki Matsumoto, Tomoyuki Noda, Toshifumi Ozaki

**Affiliations:** a Department of Orthopedic Surgery, Kochi Health Sciences Center, Kochi, Japan; b Department of Orthopedic Surgery, Kawasaki Medical School General Medical Center, Okayama, Japan; c Department of Orthopedic Surgery, Kawasaki Medical School, Kurashiki, Okayama, Japan; d Department of Orthopedic Surgery, Okayama University Hospital, Okayama, Japan

**Keywords:** Proximal humeral fracture, Late-onset neuropathy, Locking plate, Implant related complication

## Abstract

There are currently no reports of implant-related neuropathy associated with humeral proximal fracture surgery. Herein, we report a case of implant-related late-onset neuropathy that developed 3 years after proximal humeral fracture surgery.

A 51-year-old man underwent minimally invasive plate osteosynthesis for a left proximal humeral fracture 3 years prior. Left upper limb pain and reduced angle of elevation of the shoulder were recognized 1 month before the outpatient consultation. Numbness was noted on the ulnar side of the hand, and radiating pain to the ulnar nerve region was noted during shoulder abduction and compression of the medial side of the upper arm. Computed tomography revealed close proximity of the neurovascular bundle to the locking screw along with muscle atrophy around the shoulder. Hence, the patient was diagnosed with neuropathy. After implant removal, the pain in the ulnar nerve region improved, and the upper arm could be elevated.

In our case, the cause of muscle atrophy was axillary nerve manipulation and cervical myelopathy caused by the operation. When late-onset neuropathy occurs, implant-related neuropathy with muscle atrophy should be considered.

## Introduction

There are several reports on complications associated with humeral fracture surgery. For example, intra-articular penetration or loosening of the locking screw in proximal humeral fractures [1], [2] and ulnar nerve neuropathy are associated with ulnar nerve detachment during distal humerus fracture surgery [3]. However, reports of neuropathy caused by implants for proximal humerus fractures are rare. We report a rare case of a neurological disorder caused by a proximal humeral implant 3 years after proximal humeral fracture.

Informed consent to publish the case report was obtained. This report does not contain any personal information that could lead to the identification of the patient.

## Case

**Fig. 1:**
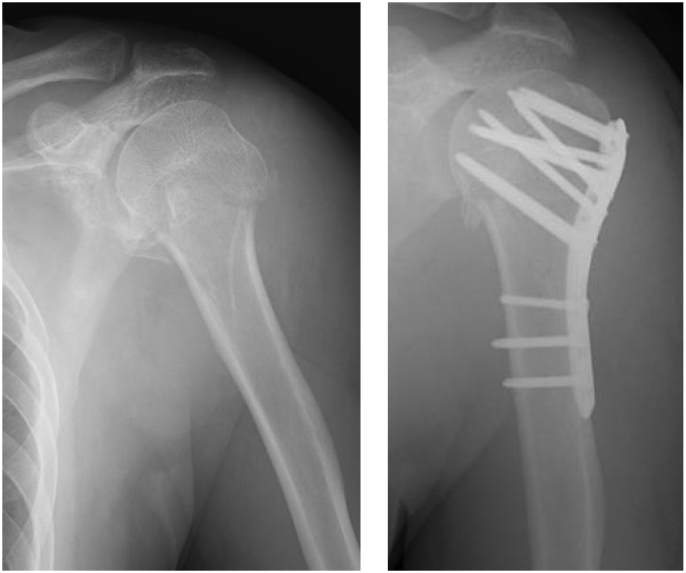
Radiographs: Proximal humeral fracture, pre and post-surgery.

**Fig. 2:**
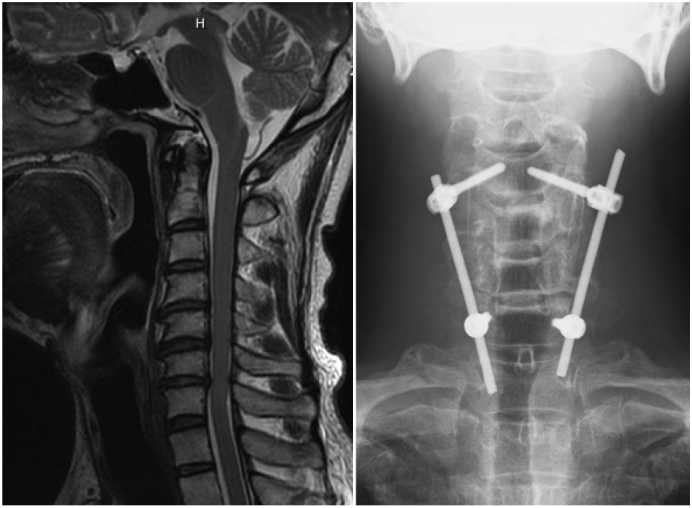
Preoperative magnetic resonance imaging and postoperative radiograph of cervical spine.

The patient was a 51-year-old man who had undergone osteosynthesis with a locking plate using the minimally invasive plate osteosynthesis method for a proximal humeral fracture at our hospital 3 years ago (Fig. 1). Bone union was achieved without problems within 3 months after the surgery. One year later, the patient experienced numbness in the upper left limbs and was diagnosed with cervical myelopathy due to posterior longitudinal ligament ossification of the cervical spine. Therefore, the patient underwent cervical laminoplasty and posterior fixation for cervical myelopathy (Fig. 2).

**Fig. 3:**
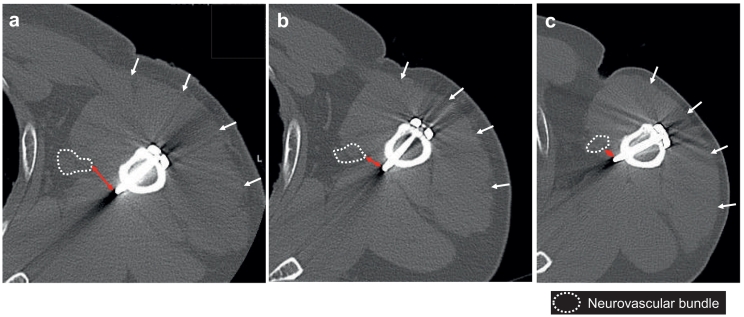
Postoperative computed tomography images of the shoulder at the level of the 2nd distal locking screw. a: One week after surgery of proximal humeral fracture. b: Three months after surgery. c: Three years after surgery. white arrow: edge of the deltoid muscle. Red arrow: distance between the neurovascular bundle and the tip of the screw. (For interpretation of the references to colour in this figure legend, the reader is referred to the web version of this article.)

**Fig. 4:**
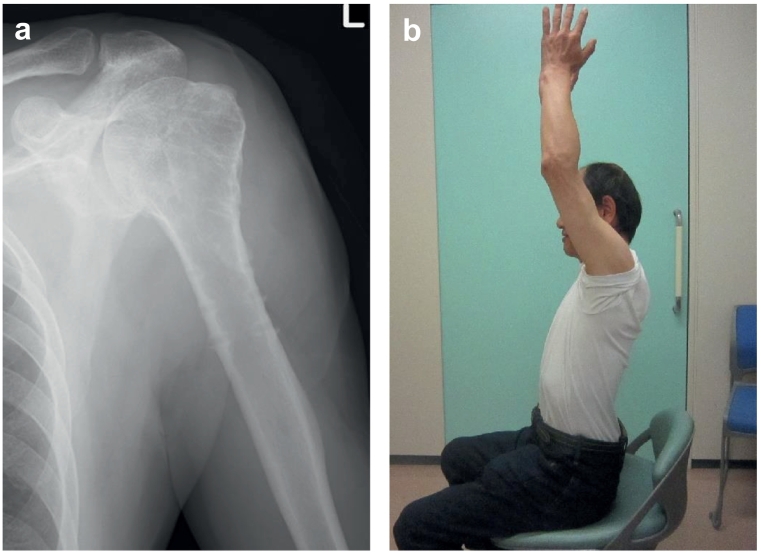
a: Postoperative shoulder radiograph after screw and plate removal b: Shoulder elevation by the patient 3 months after screw removal.

The patient experienced discomfort even after the operation and complained of pain 1 month before the consultation. At the time of consultation, it was difficult to extend the left hand and wrist joint, and the anterior elevation of the left shoulder was limited to 30° due to pain. Numbness was noted on the ulnar side of the hand, and radiating pain to the ulnar nerve region was noted due to shoulder abduction and compression of the medial side of the upper arm. A plain radiograph of the left shoulder revealed bone union at the fractured site, and no particular abnormality was found in the implant. However, postoperative computed tomography showed progressive muscular atrophy, and it was confirmed that the distal locking screw and the neurovascular bundle have moved closer to each other over time (Fig. 3). Although the patient had no abnormalities in nerve conduction velocity, he was diagnosed with neuropathy due to the locking screw; hence, removal of the plate and screw was performed (Fig. 4a). Three months after the operation, muscle spasm due to stimulation of the medial upper arm remained; however, the fingers could be extended, and the anterior elevation angle of the left shoulder improved from 30° to 160° (Fig. 4b).

## Discussion

Implant-related complications of proximal humeral fractures include screw perforation, cutout, loosening, retraction, and subacromial impingement [1], [2]. As for neuropathy associated with humeral fractures, several studies report radial nerve palsies in humeral shaft fractures [4] and ulnar nerve disorders associated with ulnar nerve detachment in distal humerus fractures [3]. Few studies have reported on neuropathy associated with proximal humeral fractures. Veilleux et al. showed that median nerve and radial nerve palsies were associated with fractures of the proximal humerus [5]. Sungelo et al. reported on a case of late-onset radial nerve palsy due to a pseudo-aneurysm that developed 9 weeks after proximal humeral fracture [6], and Kazimoglu et al. reported a case of late-onset radial nerve injury associated with pin migration after a proximal humeral fracture [7]. However, to our knowledge, there are currently no reports on late-onset neuropathy associated with proximal humeral fracture implants, and our study is the first report this.

In the present case, neuropathy was caused by the proximity of the neurovascular bundle to the tip of a screw triggered by muscular atrophy. Muscular atrophy can be caused by axillary neuropathy during surgery for proximal humeral fractures [8] and cervical myelopathy progression [9]. Laminoplasty and posterior spinal fixation were performed 2 years before onset of radiation pain and limitation of shoulder elevation; hence, it is possible that cervical myelopathy developed as a result, causing muscular atrophy. If upper limb neuropathy is observed after surgery for proximal humeral fractures, it is necessary to consider the possibility of muscular atrophy caused by cervical myelopathy. In addition, it is clear that the upper limb pain observed in our case was not associated with cervical myelopathy as the symptoms improved promptly after the implant was removed.

No particular abnormality was found in the nerve conduction velocity test; however, it is possible that the operation was performed before the neurovascular bundle and the screw were in complete contact and irreversible nerve disorder occurred. However, compression of the medial part of the upper arm along with elevation and abduction of the shoulder caused the neurovascular bundle to come into contact with the screw, inducing symptoms.

## Conclusion

We report a case of implant-related neuropathy that developed due to muscular atrophy 3 years after surgery for proximal humeral fracture. If upper limb neuropathy is observed postoperatively, it is necessary to consider the possibility of neuropathy caused by implants.

## Declaration of competing interest

None.
